# Measuring Raven’s Progressive Matrices Combining Eye-Tracking Technology and Machine Learning (ML) Models

**DOI:** 10.3390/jintelligence12110116

**Published:** 2024-11-13

**Authors:** Shumeng Ma, Ning Jia

**Affiliations:** College of Education, Hebei Normal University, Shijiazhuang 050025, China

**Keywords:** machine learning, Raven’s progressive matrices, eye-tracking technology

## Abstract

Extended testing time in Raven’s Progressive Matrices (RPM) can lead to increased fatigue and reduced motivation, which may impair cognitive task performance. This study explores the application of artificial intelligence (AI) in RPM by combining eye-tracking technology with machine learning (ML) models, aiming to explore new methods for improving the efficiency of RPM testing and to identify the key metrics involved. Using eye-tracking metrics as features, ten ML models were trained, with the XGBoost model demonstrating superior performance. Notably, we further refined the period of interest and reduced the number of metrics, achieving strong performance, with accuracy, precision, and recall all above 0.8, using only 60% of the response time and nine eye-tracking metrics. This study also examines the role of several key metrics in RPM and offers valuable insights for future research.

## 1. Introduction

Raven’s Progressive Matrices (RPM) is a non-verbal intelligence test and is considered an effective tool for measuring Spearman’s g factor (Raven 1941; Vernon and Parry 1949). This test has gained widespread application due to its simple and easily interpretable results. As a non-verbal test, RPM holds particular advantages in cross-cultural research, as it can be used by individuals from different language, professional, and cultural backgrounds. The RPM has found widespread use across various fields, including educational settings, organizational management, military, and clinical diagnostics. For instance, it is employed to assess students’ cognitive abilities in educational settings (Antoniou et al. 2022; Mert et al. 2016; Paz-Baruch and Maor 2023) and to evaluate employees’ problem-solving skills in organizational management (Klein et al. 2015). In military settings, the test is used to assess the capabilities of new recruits (Vanderpool and Catano 2008) and the neurocognitive traits of young enlistees (Theleritis et al. 2012). Due to its excellent performance in assessing cognitive abilities and detecting cognitive impairments, the RPM is also widely used in clinical diagnostics, such as in the assessment of mild cognitive impairment and Alzheimer’s disease (Angiolillo et al. 2023; Yoshiura et al. 2011).

With the advancement of technology, psychological testing has continued to evolve. The goal of psychometrics has always been to ensure testing is scientific, efficient, and accurate. With the development of artificial intelligence, more and more tests have adopted computer-aided testing. This both simplifies the testing process and improves the validity and accuracy of psychological tests. For example, through machine learning, we can fit data to more appropriate models and deploy these trained models in real-world scenarios to achieve more accurate predictions (Hatton et al. 2019; Wang et al. 2023a).

However, research on the RPM lags behind the development of AI technologies like machine learning. Currently, only a few studies have applied machine learning methods to RPM, mainly aimed at reducing the test length, which lags behind the deeper integration of AI in other tests. This study aims to advance this research direction. Given the significant role of visual information in cognitive processing (Hering 1879; Wells 1792), this study combines eye-tracking technology with machine learning models to advance the RPM applications of computer technology.

Considering that in educational and developmental studies, general cognitive ability, as measured by the RPM, is typically regarded as a background variable rather than a variable of interest (Cheung et al. 2016; Meinhardt-Injac et al. 2020), the inclusion of such tests significantly extends the duration of test administration. This extension poses a potential issue, as participants have limited attention spans and time for research participation. Consequently, the assessment of general cognitive ability might substantially interfere with the measurement of variables of interest. Therefore, we explored the performance of machine learning models in predicting the accuracy of responses on a matrix reasoning test, aiming to assess the feasibility of combining machine learning with eye-tracking technology to reduce test duration. By collecting multimodal information and applying machine learning algorithms for effective prediction, we aim to achieve the goal of shortening the testing time.

### 1.1. Raven’s Progressive Matrices

The RPM is a non-verbal test designed by the British psychologist John C. Raven based on the two-factor theory of intelligence. It primarily measures an individual’s reasoning ability within the general factor (Raven 1941). Since RPM is a non-verbal intelligence test, it is less susceptible to interference from factors such as educational level and cultural differences. Over time, its reliability and validity have been verified across a wide range of populations (Raven 2000; Van der Ven and Ellis 2000).

The RPM consists of 60 items, divided into five increasingly difficult sets: A, B, C, D, and E. Each set contains 12 items, arranged in increasing order of difficulty. Each item consists of a large image with a part missing from the lower right corner, and the task for the examinee is to discover the pattern or rule based on the relationships within the large image and determine which small image best completes it.

With advances in technology and growing demand for psychological testing, the RPM has revealed several limitations. Researchers have questioned the accuracy of this test, especially the effect of task fatigue (Kramer and Huizenga 2023). Developing shorter versions of the RPM is one solution. For example, Langener et al. (2022) reduced the test to 15 items. The shortened version has been shown to effectively replace the original while significantly reducing participant fatigue (Kramer and Huizenga 2023). However, this approach may result in the loss of certain information from the original test, such as the effect of increasing difficulty. Therefore, this study explores the feasibility of an alternative solution. By combining computer technology, we utilize eye-tracking information and machine learning algorithms to achieve accurate predictions with shorter response times, thereby enhancing the efficiency of the test.

### 1.2. Application of ML in Psychological Testing

Artificial intelligence (AI) explores and develops methods, techniques, and applications to simulate and extend human intelligence. AI is widely applied in fields like healthcare, finance, and entertainment. Machine learning, a key branch of AI, offers significant advantages in handling high-dimensional data over traditional linear models (Geng et al. 2015). It extracts nonlinear and seemingly unrelated factors that are difficult to identify using traditional methods (Doyen and Dadario 2022; Wu et al. 2020). With its strong capabilities, machine learning is widely applied across various fields (Deo 2015).

In recent years, AI, especially machine learning, has gradually been applied to the field of psychology, bringing about innovations in psychological assessment methods and giving rise to a new field known as “psychometric AI.” In this field, the use of AI algorithms for test scoring has been widely explored (Chen et al. 2010; Hussein et al. 2019). These studies have shown that AI scoring is highly consistent with human raters, demonstrating high reliability. This indicates that integrating AI with psychometrics can not only simplify the assessment process but also improve the accuracy, efficiency, and effectiveness of measurements. More importantly, applying AI to psychological assessments helps reduce the influence of human subjective factors, thereby enhancing the objectivity of the tests (Aldhyani et al. 2022). With the widespread application of machine learning algorithms, researchers have extensively explored the application of artificial intelligence in psychological testing, demonstrating that using machine learning methods to measure complex psychological traits (such as abilities and personality) is effective. For instance, Wu et al. (2015) predicted personality traits for over 70,000 participants using machine learning algorithms. The results indicated that machine learning technology can be more accurate than humans in assessing personality traits. Additionally, AI has also proven effective in the measurement of abilities, with relevant research primarily focusing on predicting academic performance. For example, Abuzinadah et al. (2023) successfully predicted students’ online learning performance using multiple machine learning models, including the Extra Tree Classifier. Ojajuni et al. (2021) employed various machine learning algorithms to predict learning outcomes, achieving an accuracy rate of over 97%. Furthermore, Wang et al. (2023b) explored emotional intelligence using eye-tracking technology and machine learning algorithms. The combination of these two technologies has also been applied in intelligence research (Bardach et al. 2024). Bardach and colleagues used gaze patterns to build models that predicted intelligence test scores and found that a significant portion of the variance in test performance (35.91%) could be explained by gaze patterns. This highlights the important role of eye movements in understanding intelligence test performance, as well as the feasibility of using machine learning methods for prediction. Although the effectiveness of artificial intelligence in psychometrics has been widely validated, few studies have assessed the accuracy of reasoning correctness in this field. Therefore, this study aims to systematically examine the accuracy of artificial intelligence in measuring reasoning correctness.

Given the complexity of machine learning, model results are often difficult to interpret. Therefore, machine learning algorithms are sometimes referred to as “black box methods”, limiting their practical application (Lee et al. 2018; Yarkoni and Westfall 2017). To improve model interpretability, this study will use SHapley Additive exPlanations (SHAP) to analyze the importance ranking of each predictor variable in the best-performing machine learning model. Compared to other explanation methods, SHAP has significant advantages in visualizing complex ML models (Lundberg and Lee 2017a). SHAP calculates each feature’s contribution to model predictions using Shapley values. Derived from the game theory, Shapley values provide a fair method for distributing payoffs in cooperative games, ensuring that SHAP allocates each feature’s contribution fairly (Lundberg and Lee 2017; Nordin et al. 2023). SHAP offers multiple visualization tools that display feature importance and the model’s decision-making process intuitively. Additionally, SHAP is compatible with various models, including linear regression, decision trees, SVMs, and neural networks (Lundberg and Lee 2017). Therefore, SHAP has broad applicability and practical value in real-world scenarios. For example, SHAP’s effectiveness in improving model interpretability has been demonstrated in suicide risk prediction (Nordin et al. 2023) and disability risk prediction in healthy elderly individuals (Han and Wang 2023).

### 1.3. ML-Based Psychometrics Using Eye-Tracking for RPM

Eye-tracking technology can capture an individual’s eye-tracking information throughout visual tasks, making it useful for studying cognitive processes related to visual activities (Eckstein et al. 2017). Technological advancements have improved the sensitivity, accuracy, and usability of eye-trackers. Eye-tracking is increasingly used in psychological research. Beyond cognitive processes, eye-tracking is widely used in studies of interpersonal interactions (Valtakari et al. 2021) and self-esteem (Potthoff and Schienle 2021).

With the advancement of technology, some studies have attempted to combine eye-tracking technology with artificial intelligence to obtain more objective and accurate results. Given that the features obtained from eye-tracking data may interact with each other in predicting outcomes, and that not all associations between eye-tracking metrics and the target features are linear, this poses a challenge for traditional analytical methods. However, AI models are highly capable of handling a large number of eye-tracking measures (Wang et al. 2023b). For example, Berkovsky et al. (2019) successfully predicted HEXACO personality test scores using eye-tracking technology and ML algorithms, achieving 90% accuracy. Eye-tracking and machine learning have also shown advantages in predicting abilities. ML models using eye-tracking features have demonstrated excellent performance in predicting reading ability (Shi and Jiang 2024). In predicting emotional intelligence, machine learning models achieved high accuracy with just 2 to 5 s of eye-tracking data (Wang et al. 2023b).

However, there has been less exploration in reasoning abilities. Thibaut et al. (2022) explored analogical reasoning using textual materials, employing eye-tracking and machine learning methods to achieve high-probability predictions. Specifically, they used support vector machines to process the data and identified which search strategies best predicted the outcome of a trial (error or correct) or the type of analogy (simple or complex). This demonstrated the effectiveness of eye-tracking technology and machine learning algorithms in complex cognitive tasks such as reasoning. To further explore reasoning and address the gap in AI’s study of abstract reasoning abilities, this study aims to systematically examine the effectiveness of various models in predicting the correctness of responses to RPM using eye-tracking technology and machine learning models while simplifying the process as much as possible.

More specifically, this study aims to contribute to the literature on AI-based psychometrics for reasoning correctness by investigating three fundamental questions: (1) What level of accuracy can machine learning models achieve in measuring the correctness of abstract reasoning, and which model performs best? (2) If machine learning models can predict reasoning correctness using eye-tracking data, what are the unique eye-tracking features that best predict reasoning correctness? (3) How much data do machine learning models need to achieve high accuracy in measuring reasoning correctness? Specifically, is it possible to accurately measure reasoning correctness using fewer indicators and less data over a shorter period? By exploring these three fundamental questions, we attempt to find a way to improve the efficiency of the test.

## 2. Materials and Methods

### 2.1. Participants

The sample consisted of 50 students (24% female) from a university in Hebei, aged 19 to 23. This study was approved by the university’s Institutional Review Board. Upon completing the experiment, the participants received monetary compensation in exchange for their participation.

### 2.2. Experimental Materials and Procedure

The experimental material was Raven’s Standard Progressive Matrices (RSPM), the most commonly used version, consisting of the initial 60 items suitable for all age groups. Each item features a geometric pattern with a missing piece, and participants must choose the correct option to complete the pattern. The test includes five sets (A, B, C, D, E), each containing 12 items, with increasing difficulty within each set. Originally published in 1938, the test is considered a valid indicator of general cognitive ability worldwide (Raven 2000).

The experiment was conducted in an eye-tracking laboratory using the Eyelink1000Plus system to record monocular eye movement data. Before the experiment, instructions were given advising the participants to keep their head position as still as possible. After a 9-point calibration, the experiment commenced. The RSPM items and options were presented simultaneously, and the participants selected their answers using a mouse. Once an answer was selected, they moved on to the next item. The stimuli were displayed on a 17-inch monitor with a resolution of 1024 × 768, positioned 60 cm away from the participants, providing a visual angle of 4.1°. The test items were presented in sequence, and the participants made their choices by clicking directly on the corresponding pattern with the mouse, with a time limit of 5 min per item. The mouse cursor was set as a solid red circular shape with a diameter of 0.5 cm. When each item was presented, the cursor was positioned at the center of the screen.

During the inter-trial interval, a calibration screen appears with a black fixation point at the center. The task will be presented only after the participants fixate on the black dot and press the spacebar, ensuring that their gaze is centered on the screen when viewing the task. Considering the potential for visual fatigue due to prolonged screen exposure, the participants are informed that they can close their eyes for a brief rest when the calibration screen appears (without moving their heads). After every 12 trials, a longer break will be provided.

### 2.3. Eye-Tracking Data Metrics

In this study, we primarily selected fixation count, fixation duration, saccade count, and saccade duration as features for the machine learning models.

Fixation count and fixation duration reflect the participants’ information extraction and comprehension. Generally, a higher fixation count or longer fixation duration indicates a better understanding of the information. However, an excessively high fixation count or prolonged fixation duration may suggest difficulties in information acquisition, whereas too few fixations or short fixation duration may indicate insufficient information acquisition (Fu et al. 2013; Pan et al. 2009).

Saccade count refers to the number of movements from one fixation point to another. There are relatively stable fixations between saccades; more saccades indicate a longer search process by the participant. In the context of RPM, the saccade count reflects the participant’s perception of the complexity of the items. An excessively high saccade count indicates that the participant is unable to effectively identify the underlying patterns in the task; while too few saccades indicate an insufficient information search (Silva et al. 2020).

Saccade distance refers to the distance between two consecutive fixation points and reflects the participant’s perceptual span. A larger saccade distance indicates that the participant is acquiring a larger amount of information in a single fixation. In the context of RPM, the saccade count can reflect the participant’s reasoning efficiency, with a larger saccade distance indicating higher reasoning efficiency (Petrov et al. 2011; Schweizer 1998).

Overall, eye movement information can index underlying cognitive processes. Considering that machine learning can analyze large amounts of eye movement data, we have taken into account various eye movement metrics as comprehensively as possible, including information from the initial, first, second, and final rounds of eye movements.

**Figure 1:**
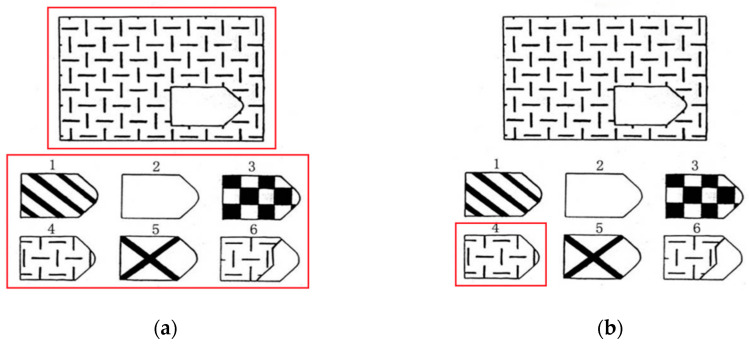
Areas of interest (AOIs) set in Raven’s Progressive Matrices (using the first item as an example). The left image (**a**) shows “answers” and “correct”, while the right image (**b**) shows “correct”.

This study selected three areas of interest. The large image in the question part was designated as “question”, all the small images in the options part were designated as “answers”, and the small image representing the correct answer was designated as “correct”. Due to the overlap between the “answers” and “correct”, we extracted the eye-tracking metrics for “correct” separately (Holmqvist et al. 2011) (see Figure 1 for an example). For each AOI, we extracted fixation count, fixation duration, first fixation duration, second fixation duration, and other metrics.

### 2.4. Data Analysis

Data analysis comprised three main steps. The first step was data preprocessing. Specifically, this step involved creating areas of interest (AOIs) and periods of interest (POIs), followed by standardizing the data. The second step was model building. In this step, eye-tracking data were used as features in various models for training, and the best-performing model was selected. The third step was data reduction. Specifically, the models were built sequentially according to POIs from smallest to largest to identify the minimum POI where the model performance stabilized. Next, SHAP was used to obtain the feature importance ranking for this POI. Finally, features were entered into the model in descending order of importance to select the model that achieved stable metrics.

#### 2.4.1. Data Preprocessing

This study used Data Viewer and Python 3.8 to process the eye-tracking data. First, we selected the question part, the options part, and the correct option part as the AOIs for this study. To determine the duration of eye-tracking data needed for model performance stabilization, we divided the time taken to complete each question into ten equal segments, creating ten POIs, i.e., the first 10%, first 20%, up to 100%. After creating the AOIs and POIs, we generated key eye-tracking measurement methods for the cognitive task. Subsequently, the data were standardized for the subsequent analysis.

#### 2.4.2. Machine Learning Models

After processing the data, we proceeded to run ten machine learning models: K-Nearest Neighbors (KNN), Naive Bayes (NB), decision tree (DT), Logistic Regression (LR), support vector machine (SVM), Random Forest (RF), Gradient Boosting, Adaptive Boosting (AdaBoost), Extreme Gradient Boosting (XGBoost), and Multilayer Perceptron (MLP). To analyze and compare these models, we followed the method of Kim et al. (2022), with all the other parameters of the machine learning models set to default except for the necessary adjustments. More details about the models can be found in Supplementary Table S1.

To objectively compare each machine learning algorithm and reduce overfitting, we employed 10-fold cross-validation. This involved evenly dividing the sample into ten mutually exclusive parts and conducting ten training sessions. In each session, nine parts were used as the training set, and the remaining part was used as the validation set. The final metrics were the average values of the model’s performance on the test set after the ten training sessions. Balanced accuracy, precision, recall, and the area under the curve (AUC) were the metrics used to evaluate the performance of a classification model. Accuracy measures how well a model correctly identifies both positive and negative instances, calculated by dividing the total number of correct predictions (true positives and true negatives) by the total number of instances. Precision focuses on the proportion of correctly predicted positive instances among all the instances predicted as positive. It is determined by dividing the number of true positive predictions by the sum of true positives and false positives. Recall, also known as sensitivity or the true positive rate, assesses the proportion of actual positive instances that the model correctly identifies. Balanced accuracy adjusts for class imbalance by calculating the average of sensitivity (recall) and specificity, offering a more reliable performance measure when class distributions are uneven. The AUC evaluates the area under the receiver operating characteristic (ROC) curve, providing a comprehensive assessment of the model’s ability to distinguish between classes.

Given the class imbalance, we adopted the modeling approach of Zhang et al. (2023), which involves applying the SMOTE method for upsampling. Each index value was obtained by performing a 10-fold cross-validation on the balanced samples generated by SMOTE. Additionally, recognizing the high variability that can occur in machine learning model results, we repeated the modeling process 10 times using different random seeds and averaged the outcomes to ensure robustness.

#### 2.4.3. Shapley Additive Explanations (SHAP)

Given the limited interpretability of machine learning results, we do not clearly understand how various features contribute to predicting reasoning correctness. To identify which eye-tracking metrics play more significant roles, we used the SHAP method to interpret the best-performing black-box model. SHAP estimates the impact of each feature on the outcome based on game theory principles, enhancing model interpretability by calculating the Shapley values to measure the contribution of each feature as its importance (Lundberg and Lee 2017). Due to its advantages in interpretability and visualization, SHAP is widely used in the machine learning field (Nordin et al. 2023; Wang et al. 2023a).

#### 2.4.4. Identifying the Required Data Amount

To determine whether the amount of data affects the accuracy of ML models and to ensure that the models use the minimum amount of data while maintaining accuracy, we controlled the data amount from two aspects: the number of features and the period of interest (POI). Specifically, we incrementally increased the POI from 10% to 100%. This approach allows us to determine how much eye-tracking information is needed to accurately predict the correctness of the answers in the RPM. Then, based on the identified POI, we used the feature importance derived from SHAP. By incrementally adding features to the model in order of importance, we identified the minimum number of features required for the model to achieve stable performance and selected these features accordingly.

## 3. Results

### 3.1. Descriptive Statistics

In this study, the average accuracy across all the questions was 82.05%, with the lowest accuracy for any single question being 14.58% (i.e., the percentage of participants who answered that particular question correctly). The participants’ average score was 49.23, with the lowest individual score being 31. Overall, the participants performed well. For college students, although there were some challenging questions, the overall difficulty of the test was moderate.

### 3.2. Performance of Machine Learning Models

Table 1 shows the performance of the ten machine learning models selected in this study for predicting the correctness of the RPM. All the models achieved an AUC value greater than 0.8, indicating the feasibility of using eye-tracking metrics to construct machine learning models for predicting the correctness of RPM answers. Among these, XGBoost demonstrated the best performance, with accuracy, precision, recall, and AUC all exceeding 0.90. Therefore, this study used the XGBoost model for further exploration.

### 3.3. Identifying the Required Data Amount Using ML Models

#### 3.3.1. Effect of POI Variation on Model Performance

**Figure 2:**
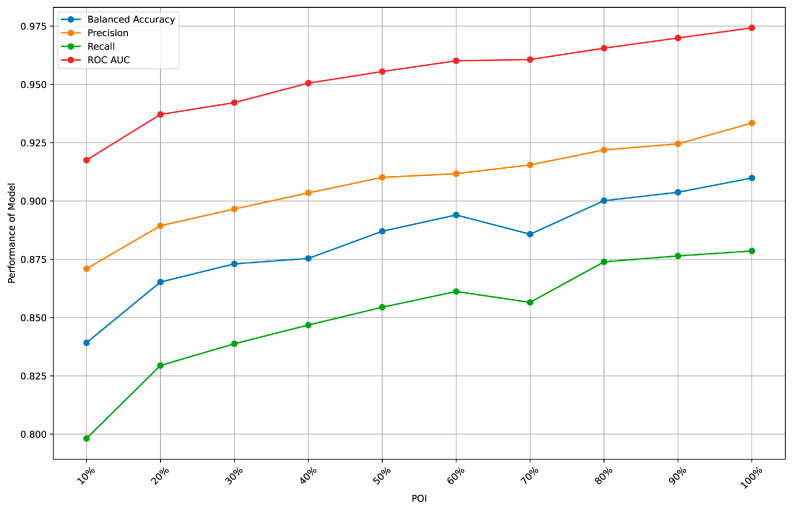
Model performance across different POIs.

To determine whether the size of the POI affects the accuracy of ML models based on eye-tracking metrics, we conducted repeated analyses using ten POIs (i.e., 10% to 100%). The analyses were performed using XGBoost, identified as the best-performing ML model in this study. To ensure the objectivity of model performance and avoid overfitting, we employed ten-fold cross-validation. The results are visualized and presented in Figure 2.

We observed that as the time window increases, the model’s overall performance improves and has not yet reached its maximum potential. Notably, when the POI constitutes 50% of the total time, all the performance metrics of the model exceed 0.85. This suggests that the model can effectively predict the answering performance with high accuracy, even when using only 50% of the available time. Considering that the model results may exhibit some degree of instability, despite the various measures we have implemented to mitigate this issue, we have conservatively chosen to use 60% as the threshold in our subsequent analysis to ensure robustness.

#### 3.3.2. Predictive Performance of Various Eye-Tracking Metrics

**Figure 3:**
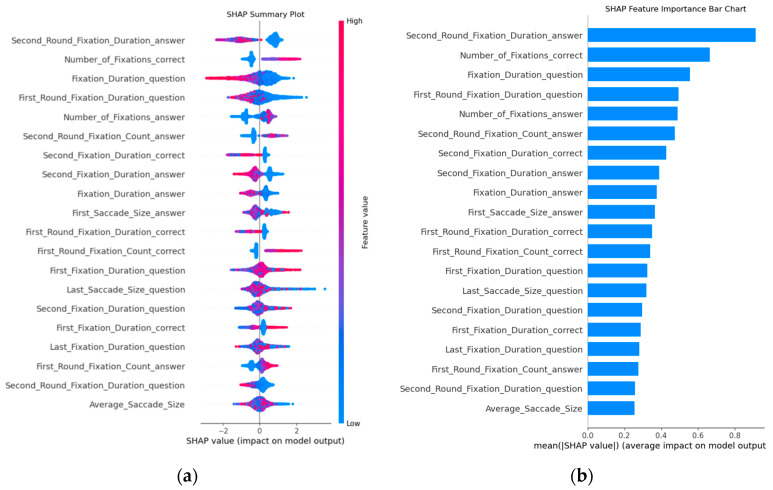
Local explanation summary (averaged feature importance) for the XGBoost. On the left, the SHAP summary plot is presented (**a**), while on the right, there is the average absolute SHAP value indicates the feature contribution (**b**).

To further investigate which eye-tracking metrics best predict the correctness of the RPM answers, we continued to use the XGBoost model because it was the best performer. By calculating the average SHAP values at the 60% point of interest (POI), we interpreted and compared the impact of the features. SHAP can visually show the contribution of each feature to a single prediction (Peng et al. 2021; Ward et al. 2021). Figure 3a displays the SHAP values of 20 features in assessing the correctness of the RPM answers. The SHAP values are on the horizontal axis, indicating how each feature affects the model’s outcome. In each feature importance row, the red and blue dots represent the correct and incorrect answers, respectively.

Figure 3b shows the important features in this model, with the vertical axis representing the ranking of feature importance. The results indicate that the duration of the second fixation in the answer area, the fixation count in the correct answer area, and the fixation duration in the question area play significant roles in predicting the correctness of the RPM answers. Based on this result, we can identify the eye-tracking metrics that play a significant role in predicting the accuracy of the responses on Raven’s Progressive Matrices, providing a foundation for the subsequent feature selection.

#### 3.3.3. Effect of Feature Quantity on Model Performance

**Figure 4:**
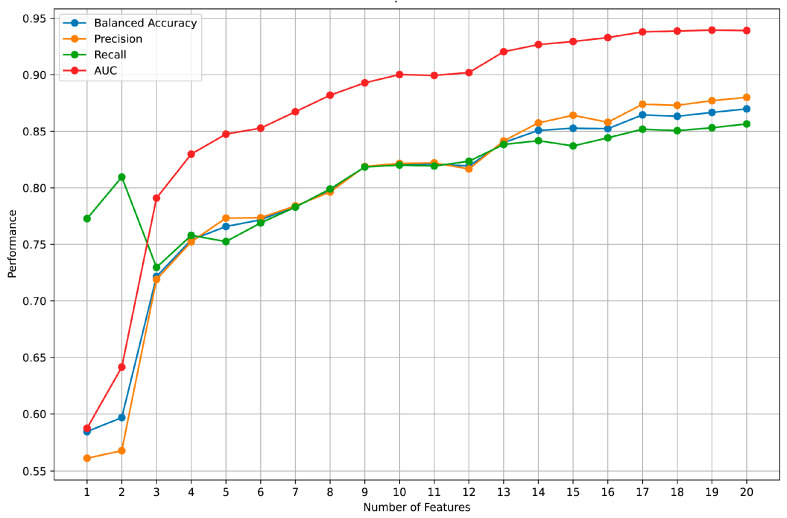
Model performance across different number of features.

Using eye-tracking information at the 60% POI, we added features to the model in descending order of importance based on their significance. The relationship between the number of features and model performance is shown in Figure 4. The results indicate that with only nine eye-tracking metrics, the model’s performance metrics can all exceed 80%. When the number of features reaches 13, further increasing the number of features results in a slow improvement in the model performance. This suggests that with 13 features, the model has nearly reached its performance limit given the current data and feature set. Therefore, we believe that in our study, using only nine eye-tracking metrics can achieve good predictive results. When the number of metrics increases to 13, further 3increasing the metrics will raise the computational cost of the model, but the improvement in model performance is very limited.

## 4. Discussion

The RPM, a classic intelligence test, has been widely applied in many fields. With the rapid development of artificial intelligence, the integration of AI technology and psychometrics is becoming increasingly close, giving rise to a new research field, psychometric AI, which is thriving. Previous studies have used machine learning algorithms to reduce the length of the RPM, alleviating the fatigue effect on the test’s accuracy to some extent. Given that reducing the test length might result in the loss of some content, the current study adopted a different approach. It utilized eye-tracking technology and machine learning algorithms to identify the correctness of answers at the item level while also reducing the amount of data required by the model without compromising accuracy. The results of running ten ML models indicate that ML methods can effectively use eye-tracking metrics to predict the correctness of RPM answers. The XGBoost model demonstrated the best performance, outperforming the other models in all the performance metrics except precision.

Encouragingly, this study found that AI models can achieve excellent performance using only the first 60% of the response time and nine eye-tracking metrics. Classical measurement theory posits that using more data can reduce measurement error, thereby achieving higher reliability and validity, i.e., better measurement quality. However, our findings suggest that leveraging learning algorithms can overcome the limitations imposed by data quantity on measurement quality. This result aligns with previous research findings, where it was discovered that using 2, 5, and 10 s of eye-tracking data yielded similarly high accuracy in predicting emotional intelligence (Wang et al. 2023b). From a psychometric perspective, this result implies two significant advantages. Firstly, it suggests the potential to save on resources such as samples and money required for testing. Secondly, it indicates that test administration time can be reduced, thereby mitigating the fatigue effect on test results.

Within the 60% POI, our study found that many eye-tracking metrics are highly predictive of the correctness of the responses in the RPM, particularly the second fixation duration in the answer area, the fixation count in the correct answer area, and the fixation duration in the question area. Among these, the second fixation duration in the answer area, the fixation count in the correct answer area, and the fixation duration in the question area ranked as the top three contributors. These findings have significant implications for future research.

Firstly, it is noteworthy that these three important metrics are distributed across the question area, the answer choice area, and the correct answer area. This distribution not only supports the validity of our AOI delineation but also highlights the significance of both the question area and the answer choice area as critical sources of information in the RPM. This finding is consistent with previous research. For instance, a study exploring cognitive processes in the RPM used eye-tracking technology to describe in detail how participants interact with both the question area and the answer choice area, emphasizing the role of these areas as essential information sources during problem-solving (Carpenter et al. 1990).

Future research could consider presenting the question part of the RPM first for a certain period, followed by the options. This approach would allow for the collection of eye-tracking metrics during the question presentation phase and building machine learning models for prediction to further simplify the testing process.

Secondly, we found that the second fixation duration in the answer area is highly predictive. Analyzing the SHAP information, we discovered that shorter second fixation durations in the answer area are associated with higher accuracy rates. This may indicate that longer second fixations reflect difficulties in extracting information or uncertainty in selecting the correct answer, causing participants to weigh multiple options. Eye movement patterns can reflect changes in information processing strategies, especially when second fixation durations are prolonged, where participants may become trapped in complex or inappropriate strategy choices, increasing the likelihood of errors (Petrov et al. 2011).

In textual studies, re-fixations are closely related to confidence, often indicating uncertainty and the need to gather more information (Sturt and Kwon 2018). In text tasks, re-reading to gather more information is undoubtedly beneficial for correcting errors (Paape and Vasishth 2022). However, our findings suggest the opposite for the RPM, where re-fixations do not increase the likelihood of correctness. We propose two possible explanations. First, participants might process and understand visual information quickly upon first seeing the images (Shepard and Metzler 1971), meaning that during the RPM, participants acquire enough information in their initial fixations. Thus, re-fixations do not provide additional information to resolve uncertain questions. This is similar to the findings of Gonthier and Roulin (2020). They found that thoroughly processing before searching for a match and mentally constructing the answer resulted in higher accuracy compared to using the elimination method with multiple rounds of fixation. Second, when solving more difficult problems, the cognitive load may limit the effectiveness of re-fixations (Sweller 1988), potentially hindering participants from effectively processing the information obtained during the second fixation. In RPM tasks, as the duration of the second look increases, especially during complex cognitive tasks, participants’ attention and cognitive resources may gradually become depleted. As cognitive load increases, they may become more likely to overlook clues to the correct answer, leading to a decrease in accuracy. The study by Deck et al. (2021) supports this hypothesis. They investigated cognitive load in various tasks, including logic tasks based on RPM, and the results indicated that as cognitive load increased, learners’ ability to process information decreased, thereby affecting cognitive performance. Future research could explore the reasons why second looks are ineffective at improving response accuracy.

Thirdly, a higher fixation count in the correct answer area is a good predictor of higher accuracy, indicating that participants have a clear goal orientation and go through an answer verification process while responding. A study using computer simulations to mimic human responses on the RPM found that the answer verification process is crucial for ultimately solving the problems (Zhao et al. 2023). Future research could further explore the differences in the answer verification process between correct and incorrect responses when completing the RPM.

Lastly, a shorter fixation duration in the question area predicts higher accuracy, reflecting the relationship between participants’ information extraction efficiency and correctness. Shorter fixation durations often indicate that participants are more efficient in extracting information and identifying patterns, while longer fixation durations suggest possible obstacles in information acquisition in this area (Gog et al. 2005; Just and Carpenter 1976). In the RPM, prolonged fixation in the question area may indicate that participants have difficulty identifying the underlying patterns in the questions. Future research could refine this metric to explore the range of fixation durations in the question area that represent the highest likelihood of correct responses.

To our knowledge, this is the first study to explore the RPM using ML and eye-tracking metrics. With the increasing accessibility of eye-tracking and ML technologies in academia, we believe the results of this study have significant practical implications. This research represents an innovative attempt to predict RPM outcomes using eye-tracking metrics integrating machine learning methods and eye-tracking technology. This integration enhances our understanding of the RPM. Researchers have investigated behaviors such as false positives (e.g., lucky guesses) or false negatives (e.g., careless responses) inherent in multiple-choice tests like the RPM (Antoniou et al. 2022). Our study’s findings suggest a new approach to elucidating this issue by combining eye-tracking technology and machine learning methods. Our cumulative time analysis suggests that the first 60% of response time is critical for pattern recognition in the RPM. Cognitive processing during this period can predict whether the current problem will be solved correctly. Furthermore, this study provides important eye-tracking metrics for researching intellectual activities, particularly in solving graphical reasoning problems. These metrics offer valuable insights into the application of eye-tracking technology and machine learning models in intelligence testing, promoting the development of psychometric AI.

Despite many noteworthy contributions, this study has some limitations. For instance, the results may only apply to university students. Future studies should expand to other populations for broader applicability. Although breaks were provided, the participants might still have experienced fatigue. Encouragingly, our study offers new insights into mitigating fatigue effects in the RPM. Future research can build on our findings to explore new models or identify new important metrics.

## 5. Conclusions

This study explored the application of artificial intelligence in the RPM by combining eye-tracking data. We trained ten machine learning models to predict the correctness of RPM answers. The results showed that the machine learning models could achieve our objectives, with the XGBoost model performing the best. Additionally, the results indicated that the machine learning models are robust enough to make accurate predictions using only 60% of the POI and nine eye-tracking metrics. Finally, we analyzed the important metrics derived from the SHAP method. These findings suggest that the combination of machine learning and eye-tracking data provides a promising approach to enhance the efficiency of administering the RPM, potentially improving the accuracy of the test and offering new perspectives on the application of AI in cognitive assessments.

## Figures and Tables

**Table 1:** Performance of ten models in predicting RPM correctness.

Model	Balanced Accuracy	Precision	Recall	AUC
KNN	0.85	0.96	0.72	0.94
GaussianNB	0.74	0.7	0.84	0.83
DecisionTree	0.84	0.85	0.82	0.84
LogisticRegression	0.83	0.82	0.84	0.89
SVM	0.9	0.9	0.9	0.96
RandomForest	0.92	0.94	0.89	0.98
GradientBoosting	0.88	0.88	0.88	0.95
AdaBoost	0.83	0.84	0.82	0.91
XGBoost	0.92	0.93	0.91	0.98
MLP	0.9	0.92	0.88	0.96

## Data Availability

The data and analysis code for this study can be obtained by contacting the corresponding author.

## Supplementary Materials

The following supporting information can be downloaded at https://www.mdpi.com/article/10.3390/jintelligence12110116/s1, Table S1: Model Details.
