# Supplementary material for: Measuring Raven’s Progressive Matrices Combining Eye-Tracking Technology and Machine Learning (ML) Models
Source: J Intell. 2024 Nov 13;12(11):116. doi: 10.3390/jintelligence12110116 (PMC11595889; doi:10.3390/jintelligence12110116)
Supplement: Supplementary file 1 [file jintelligence-12-00116-s001.zip › jintelligence-3142392-supplementary.pdf]

Supplementary Material

Table S1. Model Details.

Model	Parameters	Default Loss Function/Objective
KNN	n_neighbors=5, weights='uniform', algorithm='auto'	distance-based voting
GaussianNB	-	Gaussian distribution likelihood
DecisionTree	criterion='gini', splitter='best'	Gini impurity
LogisticRegression	penalty='l2', solver='lbfgs', max_iter=100	Log-loss
SVM	kernel='rbf', C=1.0, gamma='scale'	Hinge loss
RandomForest	n_estimators=100, criterion='gini'	Gini impurity
GradientBoosting	n_estimators=100, learning_rate=0.1, criterion='friedman_mse'	Friedman mean squared error
AdaBoost	base_estimator=None, n_estimators=50, learning_rate=1.0, algorithm='SAMME'	Exponential loss
XGBoost	max_depth=6, learning_rate=0.3, n_estimators=100, use_label_encoder=False, eval_metric='mlogloss'	Logarithmic loss
MLP	hidden_layer_sizes=(100,), activation='relu', solver='adam', alpha=0.0001, max_iter=200	Cross-entropy
